# A Perspective on Direct Binary Capacitance Detectors for Decision-Driven Biochemical and Lab-on-Chip Applications: A CMOS Cross-Coupled-Based Capacitance Detector

**DOI:** 10.3390/mi17080909

**Published:** 2026-07-29

**Authors:** Tayebeh Azadmousavi, Saghi Forouhi, Ebrahim Ghafar-Zadeh

**Affiliations:** 1 Department of Electrical Engineering, University of Bonab, Bonab 5551395133, Iran; 2 Department of Electrical Engineering (ISY), Linköping University, 581 83 Linköping, Sweden; 3 Biologically Inspired Sensors and Actuators, Department of Electrical Engineering and Computer Science, Lassonde School of Engineering, York University, Toronto, ON M3J 1P3, Canada; egz@yorku.ca

**Keywords:** cross-coupled-based capacitance detector, state-based decision, dynamic latch, complementary metal-oxide-semiconductor

## Abstract

Capacitive sensors implemented in complementary metal-oxide-semiconductor (CMOS) technology are widely used in lab-on-chip (LoC), biomedical, and microfluidic systems. While most capacitive sensor interfaces are designed for high-resolution capacitance quantification, many practical applications require only binary decisions, event detection, or state discrimination. In such scenarios, conventional readout architectures introduce unnecessary circuit complexity, power consumption, latency, and data-processing overhead. This paper presents a CMOS cross-coupled-based capacitance detector (CBCD) that directly converts the imbalance between a sensing capacitance and a reference capacitance into a digital output. By exploiting regenerative positive feedback in a dynamic latch architecture, the proposed detector integrates sensing, comparison, and digitization within a single stage, eliminating the need for analog amplification, analog-to-digital conversion, frequency-based readout, and external thresholding circuitry. Circuit-level simulations show the ability to detect extremely small capacitance differences, demonstrate robust operation across a wide range of input capacitances, and achieve negligible power consumption. Process-corner, noise, and Monte Carlo analyses further verify reliable operation in the presence of device mismatch and process variations. Owing to its compact structure, digital-native output, and energy-efficient operation, the proposed CBCD is well suited for decision-driven sensing applications, including droplet presence detection, bubble monitoring, threshold-based diagnostics, event detection, and time-of-evaporation (ToE) measurements. The proposed architecture provides a scalable and low-complexity front-end solution for next-generation CMOS-integrated sensing platforms.

## 1. Introduction

Capacitive sensors are widely employed in detecting changes in dielectric properties, geometry, or surface composition. In the complementary metal-oxide-semiconductor (CMOS) domain, these sensors have been extensively used for applications ranging from precise biochemical measurements to microfluidic monitoring and industrial automation. CMOS capacitive sensors enable compact integration, low-voltage operation, and compatibility with large sensor arrays, making them particularly suitable for lab-on-chip (LoC) platforms. Examples include CMOS capacitive biosensors for label-free biomolecular detection, DNA analysis, and cellular characterization and tracking [1,2,3,4]. In most of these applications, the primary objective is accurate capacitance quantification, and therefore significant effort has been devoted to improving sensitivity, resolution, linearity, and dynamic range. In these implementations, the focus is often on high-resolution capacitance measurement to quantify concentration, permittivity, or other physical parameters.

However, capacitive sensing applications differ significantly in the role that capacitance information plays in the final system output. While some systems are designed to estimate capacitance accurately and preserve quantitative information, others ultimately use capacitance information to generate threshold-based decisions, trigger control actions, or detect the occurrence of specific events. As summarized in Table 1, capacitive sensing applications can be broadly categorized into three groups: quantitative sensing systems, threshold-based diagnostic systems, and event- and state-detection systems. Table 1 compares selected applications of capacitive sensors according to the role of capacitance information in the final system output and decision-making process.

The first category comprises quantitative sensing systems, where capacitance is measured to estimate a physical, chemical, or biological parameter. Representative examples include the DNA biosensor reported in [5], the protein biosensor presented in [6], and the CMOS sensor matrix for cell characterization and tracking described in [4]. In these systems, capacitance is treated as a continuous variable, and the sensing architecture is explicitly designed to preserve quantitative information. For example, the CMOS sensor matrix in [4] transduces cell-induced electrical changes into frequency-domain signals that are subsequently used for localization and characterization. Although downstream processing may involve classification or interpretation, the sensor architecture itself remains fundamentally quantitative because its objective is to estimate and preserve capacitance-related information rather than directly producing a binary decision. Consequently, performance is evaluated through metrics such as sensitivity, detection limit, linearity, and dynamic range.

The second category consists of threshold-based diagnostic systems. In these applications, capacitance is measured continuously but is ultimately compared with a predefined threshold to generate a binary decision. The capacitive COVID-19 immunosensor reported in [7] is a representative example, where the measured capacitance change is used to classify samples as positive or negative. Although the sensing mechanism is quantitative, the final output of the system is typically binary because it is derived from a threshold-based decision process.

The third category comprises event- and state-detection systems, where the information of interest is not the capacitance value itself but whether a particular condition exists. Examples include bubble-monitoring systems [8,9], where the objective is to determine whether a bubble is present or absent. The integrated digital microfluidic system reported in [10] uses capacitance-difference sensing to determine droplet position and routing decisions. In both cases, the final functionality relies on discrete decisions or control actions rather than precise capacitance quantification. Nevertheless, these systems typically employ continuous capacitance measurement followed by signal processing, thresholding, and control logic.

These examples illustrate that capacitive sensing applications span a continuum from quantitative measurement to decision-driven operation. While quantitative sensing systems require accurate capacitance estimation and therefore benefit from sophisticated readout architectures, many practical LoC applications ultimately rely only on discrete decisions, state transitions, or control actions. In such applications, capacitance primarily serves as a trigger for decision-making rather than as a quantity requiring precise measurement. Nevertheless, existing capacitive interfaces remain fundamentally quantitative, since they first estimate capacitance and subsequently generate binary decisions through thresholding, classification, or control logic. Consequently, although several of the applications listed in Table 1 ultimately produce binary outputs, none directly performs capacitance discrimination within the sensing interface itself. This mismatch between the sensing objective and the circuit architecture introduces unnecessary circuit complexity, power consumption, latency, and signal-processing overhead in applications requiring only binary decisions. This observation motivates the development of capacitance interfaces that directly perform state discrimination without first generating a quantitative capacitance measurement. Such architectures can reduce circuit complexity, power consumption, area, latency, and data-processing requirements while preserving the functionality required in decision-driven applications.

Regenerative cross-coupled latches are well established in dynamic comparators, sense amplifiers, and memory circuits because of their high-speed regenerative decision capability. However, in these circuits, the latch operates only after the input signal has already been converted into a voltage or current by a preceding analog front-end. Similarly, existing capacitive sensing systems employ capacitance-to-voltage, capacitance-to-current, or capacitance-to-frequency conversion before applying thresholding or regenerative decision circuits. To the best of our knowledge, no reported CMOS capacitive interface employs a regenerative latch as the sensing interface itself, allowing the sensing capacitance and reference capacitance to directly initiate the regeneration process and simultaneously produce a digital decision. Consequently, the proposed CBCD introduces a different detection architecture in which sensing, comparison, and digitization are integrated into a single dynamic stage rather than implemented as separate functional blocks.

Motivated by this need, this work proposes a Cross-Coupled-Based Capacitance Detector (CBCD) that directly converts the imbalance between a sensing capacitance and a reference capacitance into a digital output. Unlike conventional capacitive interfaces that first estimate capacitance before applying thresholding or digital processing, the proposed architecture employs regenerative positive feedback directly as the sensing mechanism, integrating sensing, comparison, and digitization within a single dynamic CMOS stage. This eliminates the need for intermediate capacitance conversion, analog amplification, analog-to-digital conversion, frequency measurement, and external thresholding circuitry. The proposed CBCD is particularly suited to decision-driven applications such as threshold-based diagnostics, bubble detection, droplet monitoring, and time-of-evaporation (ToE) measurements, where reliable state discrimination is more important than precise capacitance quantification. It should be emphasized that the contribution of this work is not the introduction of a new regenerative latch topology. Cross-coupled regenerative circuits are already well established; the novelty lies in repurposing regenerative positive feedback as the sensing interface itself, enabling the sensing capacitances to directly determine the digital output without an intermediate quantitative readout stage. This establishes a different architectural paradigm for CMOS decision-driven capacitive sensing, in which sensing and binary decision making are inherently combined within the same circuit.

In the remainder of this paper, Section 2 reviews related work, including CMOS capacitive sensor readout interfaces and a specific application for measuring droplet evaporation time, which highlights the potential future applications of the proposed detector. Section 3 introduces the proposed capacitance detector. Section 4 presents the simulation results of the proposed circuit. Finally, Section 5 discusses the proposed method, followed by the conclusion.

## 2. Related Work

To better understand how capacitive detection principles are implemented in practice, it is useful to consider these systems in terms of their functional blocks. Regardless of whether a system is designed for binary detection or high-resolution sensing, the overall architecture follows a modular structure, where each block performs a specific role in converting a physical, chemical, or biological event into a usable electrical signal. Examining the system this way clarifies how design choices—such as circuit complexity, power consumption, and noise tolerance—are tailored to the application and highlights why binary capacitive detectors can achieve robustness and scalability with simpler circuitry compared with high-resolution sensors.

**Figure 1:**
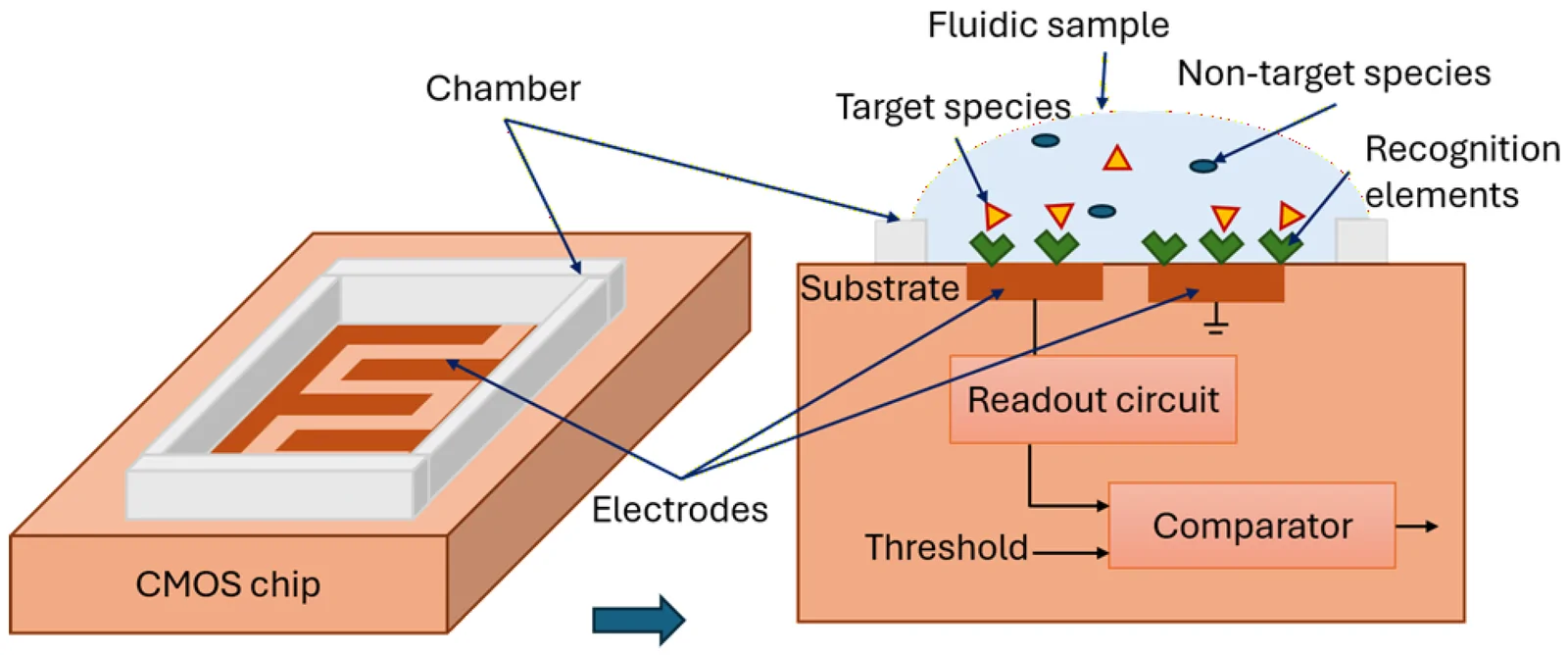
Conceptual view of an LoC-based CMOS capacitive detector.

A typical capacitive detection system in CMOS LoC platforms consists of four main blocks: (1) microfluidics or a fluidic device, (2) the sensing layer, (3) the transducer, and (4) the readout circuit and the communication system (see Figure 1).

Microfluidics or a fluidic device refers to the use of integrated microscale channels or droplets to manipulate small volumes of liquid over sensing electrodes or transducers. In simple prototypes, it can be replaced by a chamber that holds the sample on top of the sensing surface.

The sensing layer, often called the functionalized film, provides selectivity for the target analyte or event. Its composition depends on the application. For example, in biochemical sensing, antibodies, aptamers, DNA probes, or enzymes bind specific biomolecules, whereas in gas detection, polymers or metal-oxide films selectively adsorb target gases.

The transducer converts the dielectric changes from the sensing layer into a measurable capacitance change. Common transducer types include interdigitated electrodes (IDEs), parallel-plate capacitors, and MEMS-based structures.

The readout circuit encompasses the entire signal acquisition and processing chain, including the interface circuit/front-end, amplification, filtering, analog-to-digital conversion, and thresholding or decision logic. Its role is to convert the raw capacitance change into a usable electrical signal or digital output. High-resolution sensors use sophisticated analog front-ends, amplifiers, and high-resolution ADCs to preserve signal fidelity, linearity, and quantitative accuracy. The communication system transmits the processed output to off-chip systems or other on-chip modules. This can include wired digital lines, serial interfaces, or wireless transmitters depending on the application. In binary detection, a single digital line or pulse is typically sufficient, while high-resolution sensors may require multi-bit digital buses or high-speed serial interfaces to convey detailed quantitative measurements.

### 2.1. CMOS Capacitive Sensor’s Readout Interface

Although improvements in capacitive sensors are consistently boosting their performance, the detection limits and capabilities are frequently constrained by the CMOS readout interfaces. The methods used to date to read out capacitance by CMOS-based topologies mainly include oscillator-based approaches [11,12,13], lock-in detection [14] charge-sharing circuits [1,15], charge-sensitive amplifier [16], and charge-based capacitance measurement (CBCM) [17,18]. However, each of these techniques presents certain limitations.

As summarized in Table 2, charge-sharing architectures offer a simple and area-efficient solution and have been successfully employed in large sensor arrays. For example, the 16 × 16 bacterial detection array in [1] achieves a resolution of 450 aF, while the cell proliferation monitoring platform in [15] reports a resolution of 5 fF. Nevertheless, the achievable resolution is often limited by charge injection, switch nonidealities, and parasitic capacitances, which become increasingly significant when targeting atto-farad-level sensing.

Charge-sensitive amplifier-based architecture can significantly improve sensitivity and resolution. The capacitive sensor array reported in [16] achieves a sensitivity of 345 mV/fF and a resolution of 21 aF while supporting a large 320 × 320 array. However, CSA-based approaches generally require high-gain analog front-ends and careful offset and noise management, leading to increased circuit complexity, power consumption, and area overhead.

Oscillator-based capacitance-to-frequency conversion (CFC) techniques provide direct digitization of capacitance through frequency measurement and are attractive for sensor-array integration. The ring-oscillator implementation in [12] achieves an excellent resolution of 14.4 aF and a high sensitivity of 590 kHz/fF. Similarly, relaxation-oscillator-based systems have been demonstrated for dopamine detection [13] and DNA sensing [11]. However, oscillator-based approaches are inherently sensitive to process, voltage, and temperature (PVT) variations, supply noise, and phase noise, which can degrade accuracy and long-term stability.

Switched-capacitor CDC architectures, such as the SC-SAR approach reported in [19], provide direct digital outputs and good compatibility with standard CMOS technologies. However, their resolution is typically limited by quantization noise, capacitor mismatch, and SAR conversion accuracy, particularly when sensing extremely small capacitance variations.

Among the reported approaches, lock-in detection achieves the highest reported resolution. The CMOS airborne particle detection system in [14] demonstrates a remarkable resolution of 65 zF. However, this performance is obtained through highly sensitive synchronous detection techniques that typically require complex analog signal-conditioning circuits, precise reference generation, and relatively long measurement times, making large-scale array implementation challenging.

**Figure 2:**
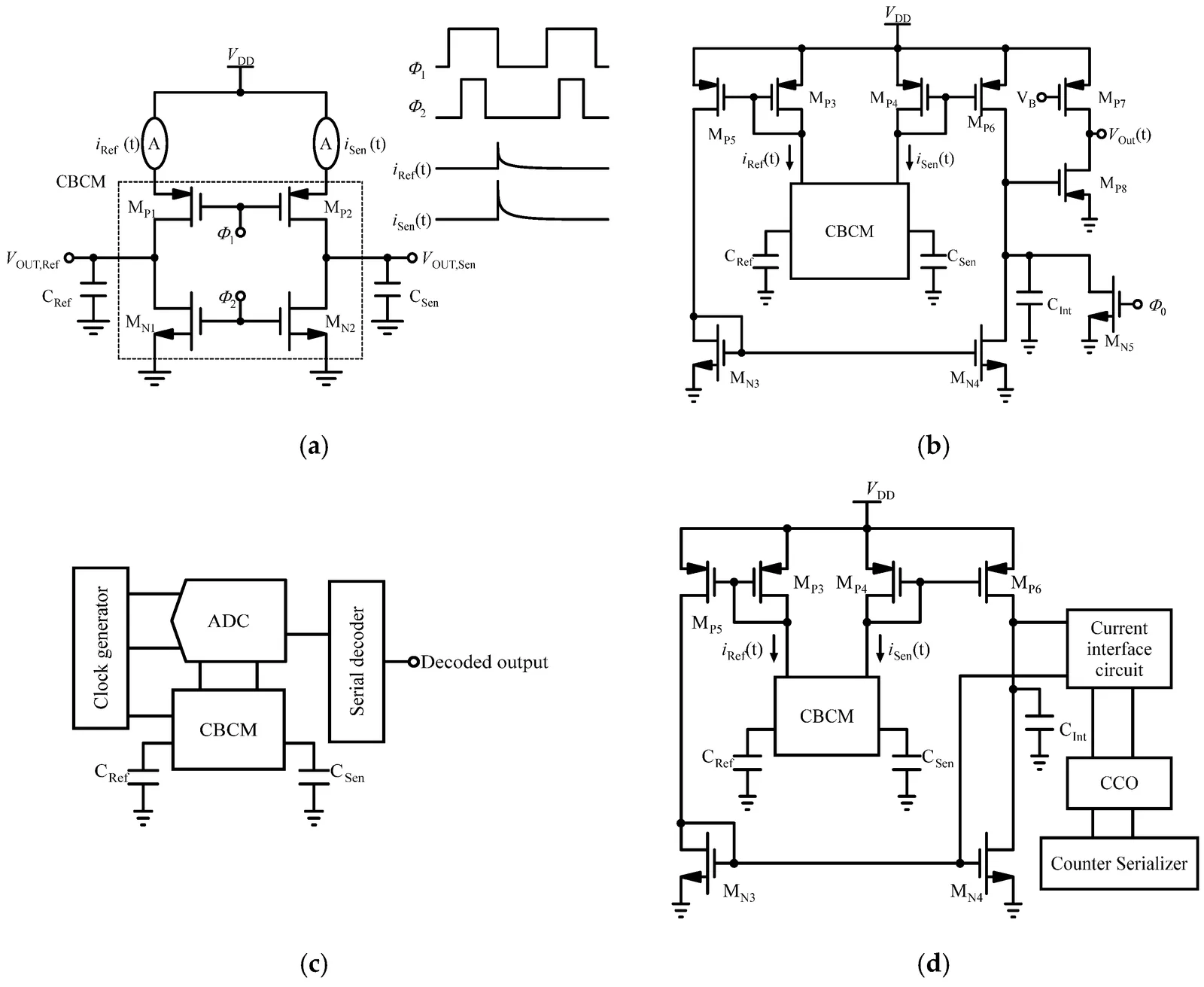
The structure of the (**a**) conventional CBCM, (**b**) Differential CBCM, (**c**) CBCM with ΣΔ modulator, (**d**) CBCM with CCO.

CBCM is one of the common topologies used in capacitive sensors as shown in Figure 2a, and it provides a compelling solution because it combines high accuracy with low complexity, making it particularly well-suited for LoC applications [21]. The core structure consists of sensing and reference capacitances and four transistors controlled by non-overlapping clock signals. To clarify its operation, when $\phi_{1}$ and $\phi_{2}$ are low, the sensing and reference capacitors are charged through the PMOS switches. In the conceptual circuit of Figure 2a, the branch currents are monitored by ideal ammeters, whereas in the practical implementation of Figure 2b, these ammeters are replaced by PMOS current mirrors. Consequently, the effective voltage excursion across the capacitors is approximately $V_{DD}-V_{th}$, where $V_{th}$ denotes the threshold voltage of the current-mirror transistors (or, equivalently, the voltage dropped across the ideal ammeters in Figure 2a). Whenever these clock pulses go high, the capacitors are discharged via pull-down switches (M_N1_ and M_N2_). As shown in Figure 2a, the currents in the two branches of this circuit, $i_{Sen}(t)$ and $i_{Ref}(t)$, are time-dependent, sharply decaying exponential currents [22]. The instantaneous currents through the two branches can be obtained by:

(1) $$i_{Sen}\left(t\right)=C_{Sen}\frac{dV_{Out,Sen}(t)}{dt}$$

(2) $$i_{Ref}\left(t\right)=C_{Ref}\frac{dV_{Out,Ref}(t)}{dt}$$

where $V_{Out,Sen}$ and $V_{Out,Ref}$ denote the instantaneous voltages across $C_{Sen}$ and $C_{Ref}$, respectively. The average of these currents throughout one period of *Φ*_1_ and *Φ*_2_ is proportional to the related capacitances and can be expressed as follows:

(3) $$I_{Sen}=\frac{1}{T_{s}}\int_{0}^{T_{s}}C_{Sen}\frac{dV_{Out,Sen}\left(t\right)}{dt}dt=\frac{C_{Sen}}{T_{s}}\int_{0}^{V_{DD}-V_{th}}dV_{Out,Sen}=\frac{{C_{Sen}(V}_{DD}-V_{th})}{T_{s}}$$

(4) $$I_{Ref}=\frac{1}{T_{s}}\int_{0}^{T_{s}}C_{Ref}\frac{dV_{Out,Ref}\left(t\right)}{dt}dt=\frac{C_{Ref}}{T_{s}}\int_{0}^{V_{DD}-V_{th}}dV_{Out,Ref}=\frac{{C_{Ref}(V}_{DD}-V_{th})}{T_{s}}$$

where $I_{Sen}$ and $I_{Ref}$ represent the average of currents $i_{Sen}\left(t\right)$ and $i_{Ref}\left(t\right)$, respectively. $T_{s}$ is the period of *Φ*_1_ and *Φ*_2_, which is the inverse of their frequency, $f_{s}$ ($T_{s}$ = ${1/f}_{s}$). Therefore, the difference between $C_{Sen}$ and $C_{Ref}$ (ΔC = $C_{Sen}-C_{Ref}$) can be obtained by subtracting the two averaged currents:

(5) $$I_{Sen}-I_{Ref}=f_{S}\left(V_{DD}-V_{th}\right)∆C$$

Thus, ΔC can be extracted with high accuracy.

Within our group’s research portfolio, several generations of CMOS CBCM interfaces have been developed for LoC and life-science applications, as summarized in Figure 2b–d. To amplify and subtract the CBCM output currents on-chip, a differential CBCM architecture was introduced, as illustrated in Figure 2b [23]. This structure converts the capacitance difference into a differential current, which is subsequently integrated by an integrating capacitor and generates an output voltage. The next generation, shown in Figure 2c, adapts a ΣΔ modulator to the CBCM interface, enabling direct digital readout while achieving sensitivities up to 250 mV/fF and a minimum detectable capacitance variation of 10 aF [17]. To improve robustness against common-mode disturbances, a fully differential core-CBCM array was developed, achieving a capacitance resolution of 10 aF and a sensitivity of 350 mV/fF [18]. More recently, current-mode wide-input-dynamic-range CBCM architectures were developed [20,24], as depicted in Figure 2d. In this approach, capacitance variations are first converted into differential currents, which are subsequently translated into frequency using a current-controlled oscillator (CCO) and digitized through frequency counting. Since the average CBCM output current is proportional to the sensed capacitance, signal integration is performed in the digital domain rather than the analog domain, thereby overcoming supply voltage limitations and significantly extending the input dynamic range. These architectures have been successfully applied to liquid-sample monitoring and droplet ToE measurements [25], achieving capacitance resolutions down to 416 aF.

Table 2 further distinguishes existing capacitive interfaces according to whether they directly perform binary capacitance detection or first estimate capacitance before applying thresholding or digital decision logic. As shown, all previously reported interfaces rely on intermediate capacitance measurement.

### 2.2. Measuring Time of Evaporation of Binary Chemical Sample Using CMOS Capacitive Sensors

Our recent work on droplet ToE measurements [25] has highlighted the importance of detecting the presence of a sample rather than performing precise quantitative capacitance measurements. Focusing on presence detection can significantly reduce circuit complexity, making it particularly suitable for low-power, small-footprint applications such as LoC systems.

The ToE measurement method presented in [25] employs a CMOS capacitive sensor composed of microscale IDEs connected to on-chip readout circuitry. The readout employs a CCO-based technique, similar to the circuit shown in Figure 2d, to monitor the presence or absence of droplets on the electrodes. When a droplet is placed on the sensor surface, the liquid alters the local dielectric environment, causing the sensor capacitance to increase. This change can be tracked over time to monitor the droplet’s behavior.

As the droplet evaporates, its volume gradually decreases. The measured capacitance remains relatively high until the remaining liquid thickness drops below a critical screen length (SL) of the electrodes. The SL depends on the electrode geometry and material, the surrounding electric field distribution, and the operating frequency. Once the thickness falls below this threshold, the capacitance decreases more noticeably, reflecting the reduced dielectric contribution from the thinning liquid layer. Small variations in liquid composition, such as changes in alcohol concentration in water–alcohol mixtures, may slightly affect the capacitance, but these changes are minor compared with the overall signal corresponding to droplet presence.

For droplets thicker than the SL, small dielectric changes may not be detectable, as the capacitance becomes largely insensitive to variations deep within the liquid. To overcome this limitation, the proposed method in [25] does not rely on measuring small dielectric changes. Instead, it quantifies the total ToE by monitoring the capacitance from the moment the droplet is placed on the sensor until it fully evaporates. This approach extends the dynamic measurement range, as ToE varies with droplet volume, while the capacitance itself may remain nearly constant for thick droplets.

In this method, ToE is defined as the total duration during which the sensor detects the presence of liquid, i.e., when the change in capacitance (ΔC) is greater than zero. Measurement begins when the droplet is introduced, producing a high initial capacitance, and ends when the liquid fully evaporates, returning the sensor to its baseline. By relying on presence-induced capacitance changes rather than precise thickness measurements, this method provides robust, accurate, and reproducible ToE measurements for droplets of various volumes and compositions. While the proof-of-concept system used a full capacitive sensor, detecting only the presence of the droplet over time is sufficient to measure ToE. This observation suggests that circuit complexity can be reduced by implementing a binary capacitance detector instead of a high-resolution sensor.

## 3. Proposed Capacitance Detector

### 3.1. Capacitive Transducer

IDEs are one of the most widely used coplanar electrode structures, especially in LoC applications. They consist of two interleaved, comb-shaped planar electrodes that can be implemented in the top metal layer of CMOS technology. Their sensing principle relies on variations in the effective dielectric constant of the medium between the electrodes.

Typically, one set of comb fingers is connected to ground, while the electrode surface is functionalized with a material—such as biological recognition layers or polymers—that is selectively sensitive to target analytes in the sample. To enhance sensitivity, the electric field between the electrode fingers can be strengthened by removing the passivation layer between them using a pad-etch process. In some cases, the passivation layer above the sensing area is also completely removed. However, aluminum (Al), commonly used in CMOS metallization, is susceptible to oxidation. When the passivation is removed and the Al electrodes are exposed to the environment, a native aluminum oxide (Al_2_O_3_) layer naturally forms upon air exposure. This thin oxide layer provides electrical insulation, protecting the underlying metal from chemical and biological interactions, while also offering a favourable surface for subsequent functionalization.

Depending on the intended sensing application, the electrochemical behaviour at the bio–electrode interface can be described using equivalent circuit models, such as the Randles model.

### 3.2. Capacitance Detector Interface Circuit

**Figure 3:**
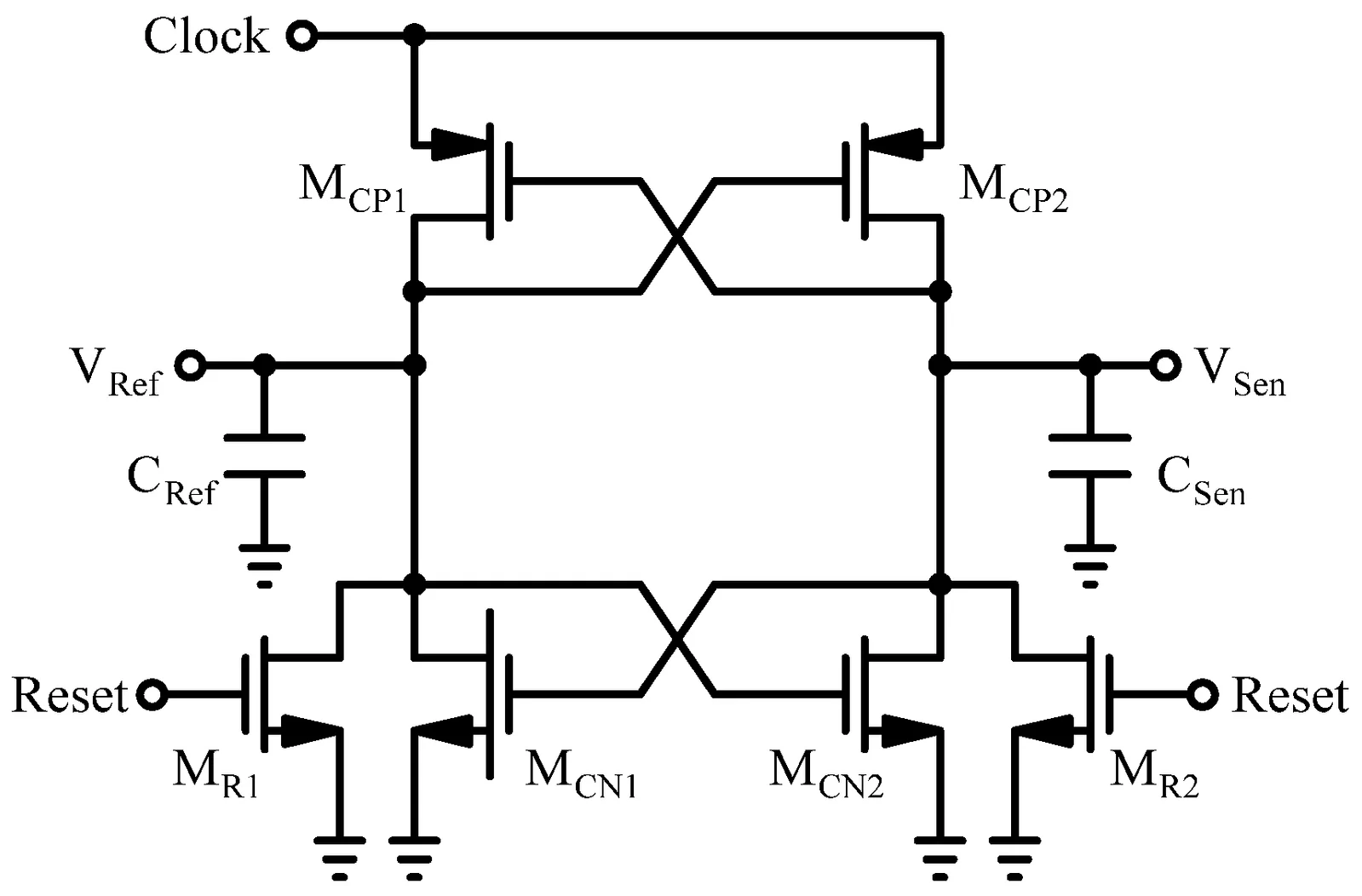
The structure of the proposed CBCD circuit.

The architecture of the proposed cross-coupled-based capacitance detector (CBCD) is shown in Figure 3. It consists of PMOS and NMOS cross-coupled pairs (M_CP1,2_ and M_CN1,2_) to simultaneously perform capacitance detection and generate a digitized output. Transistors M_Rst1,2_ act as the reset transistors to discharge the capacitances of $C_{Sen}$ and $C_{Ref}$. The clock pulse, which acts as a dynamic supply voltage, is applied inverted to the reset signal to provide dynamic detection, consequently eliminating extra power dissipation during the reset process.

**Figure 4:**
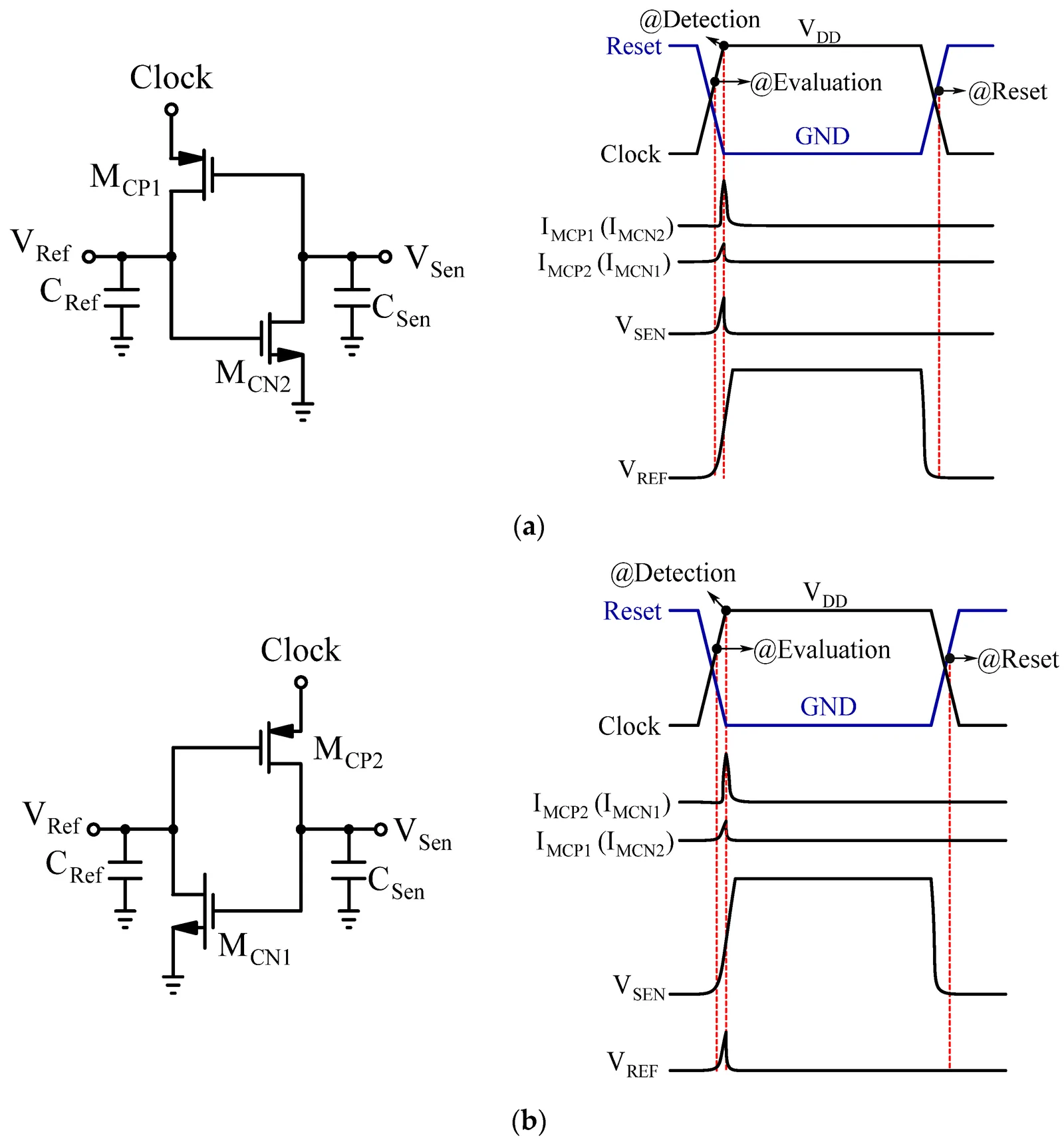
The simplified model of the CBCD with a timing diagram, which shows the process of converting the detected capacitance to a digital signal: (**a**) $C_{Sen}$ > $C_{Ref}$, (**b**) $C_{Sen}$ < $C_{Ref}$.

The capacitance detection process of the CBCD circuit comprises three operational phases: evaluation, detection, and reset. During the evaluation phase, when the clock pulse transitions above *V*_DD_/2, the cross-coupled transistors (M_CP1,2_, M_CN1,2_) are turned on, allowing the sensing and reference capacitances, $C_{Sen}$ and $C_{Ref}$, to begin charging. Consequently, the voltages at the sensing and reference output nodes, $V_{Sen}$ and $V_{Ref}$, begin to rise simultaneously from zero. Following this, the detection phase commences. The capacitance imbalance causes unequal current through the PMOS cross-coupled pair (M_CP1,2_). As illustrated in Figure 4, this imbalance causes one PMOS and one NMOS cross-coupled transistor to remain in the ON state, while the other transistors are turned off. Consequently, the node with the smaller capacitance is latched to $V_{DD}$, and the opposing node is pulled to 0 V, thereby initiating and reinforcing the latch behavior. This presents a novel and straightforward approach for capacitance detection.

As depicted in Figure 4a, when the capacitance $C_{Sen}$ exceeds $C_{Ref}$ ($C_{Sen}>C_{Ref}$), transistors of M_CP1_ and M_CN2_ are turned on, and the CBCD functions as a cross-coupled structure [26,27]. When $C_{Ref}$ exceeds $C_{Sen}$, ($C_{Sen}<C_{Ref}$), only the transistors of M_CP2_ and M_CN1_ are turned on (see Figure 4b). The inherent positive feedback of the cross-coupled configuration amplifies the initial imbalance. This action reinforces the voltage on the smaller-capacitance node and attenuates the voltage on the larger-capacitance node. Thus, the final state latches with one node at V_DD_ and the other at 0 V, producing a full digital output swing (see Figure 4). Finally, in the reset phase, M_Rst1,2_ is turned on, thereby discharging the nodes $V_{Sen}$ and $V_{Ref}$.

The cross-coupled sensing topology enables the detection of zF-level capacitance variations, making it suitable for high-sensitivity applications, such as detecting dust particles. The dynamic detection principle ensures negligible power consumption (0.45 µW). Furthermore, the merged analog and digital functions within the single-stage CBCD facilitate straightforward array implementation for scalable sensor interfaces.

## 4. Post-Layout Simulation Results

**Figure 5:**
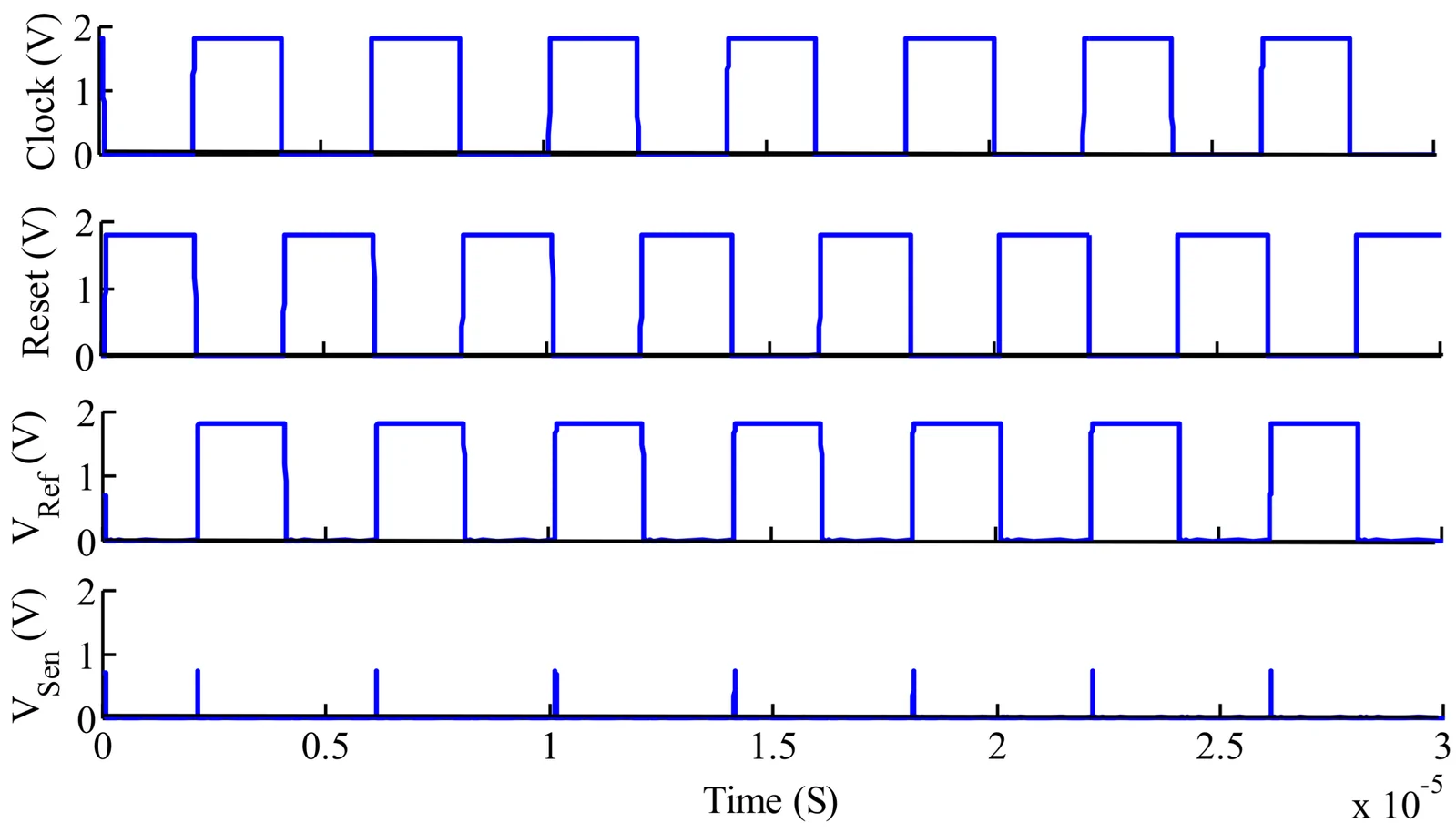
Timing diagram of capacitance detection when $C_{Sen}$ > $C_{Ref}$ (1.000001 fF > 1 fF).

This section presents the post-layout simulation results performed using Cadence Virtuoso ADE L with the Spectre simulator, employing BSIM3v3.2.4 device models for a standard 0.18-µm CMOS process (TSMC 180 nm). Nominal operating conditions are a supply voltage of 1.8 V and a temperature of 27 °C at the TT (typical-typical) process corner. Parasitic extraction was performed using Calibre Interactive PEX with R + C + CC extraction type and transistor-level mode. The circuit is driven by two non-overlapping signals (Clock and Reset) at a frequency of 250 kHz with a 50% duty cycle. The proposed design exhibits a low power consumption of 0.45 µW and provides a resolution down to the zF range. Generally, a capacitance detector detects changes in the sensing capacitance, $C_{Sen}$, which is exposed to the target, compared with a constant reference capacitance, $C_{Ref}$. The following analysis of the CBCD detector is based on simulations performed with a fixed reference capacitance ($C_{Ref}$ = 1 fF) and a variable sense capacitance ($C_{Sen}$). The sensitivity of the CBCD detector was evaluated by simulating a 1 zF capacitance mismatch, configured with $C_{Ref}$ of 1 fF and $C_{Sen}$ of 1.000001 fF. As shown in Figure 5, when $C_{Sen}$ > $C_{Ref}$, the detector results in a pulsed $V_{Ref}$ output, and $V_{Sen}$ is held at 0 V, consistent with the operating principle described in Section 2.

**Figure 6:**
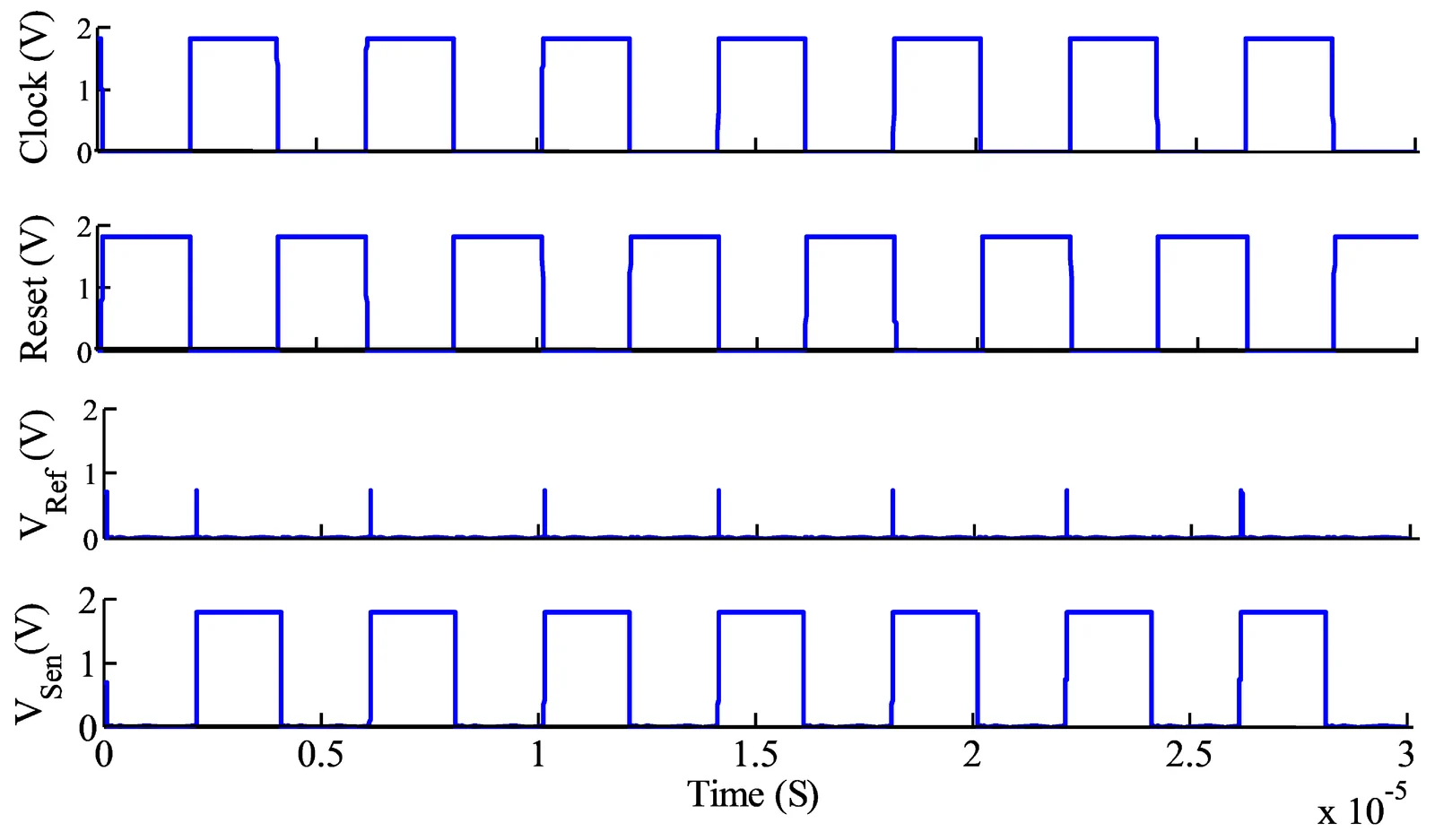
Timing diagram of capacitance detection when $C_{Sen}$ < $C_{Ref}$ (1 fF < 1.000001 fF).

Also, the timing diagram shown in Figure 6 illustrates the capacitance detection when $C_{Ref}$ exceeds $C_{Sen}$ by 1 zF. However, according to the studies conducted thus far, the cross-coupled structure has not been employed for capacitive sensing. The inherent nature of this structure enables high capacitance detection accuracy. The simulations performed herein are intended to demonstrate the high precision of the cross-shaped structure. As observed from the simulation results, the accuracy of the structure reaches the zepto-farad range. It should be noted, nevertheless, that this level of precision may be affected after fabrication due to various factors, such as parasitic capacitances and other parasitic effects, which can alter the detection accuracy. Nonetheless, the cross-coupled structure remains a promising candidate for achieving high-precision capacitive sensing.

**Figure 7:**
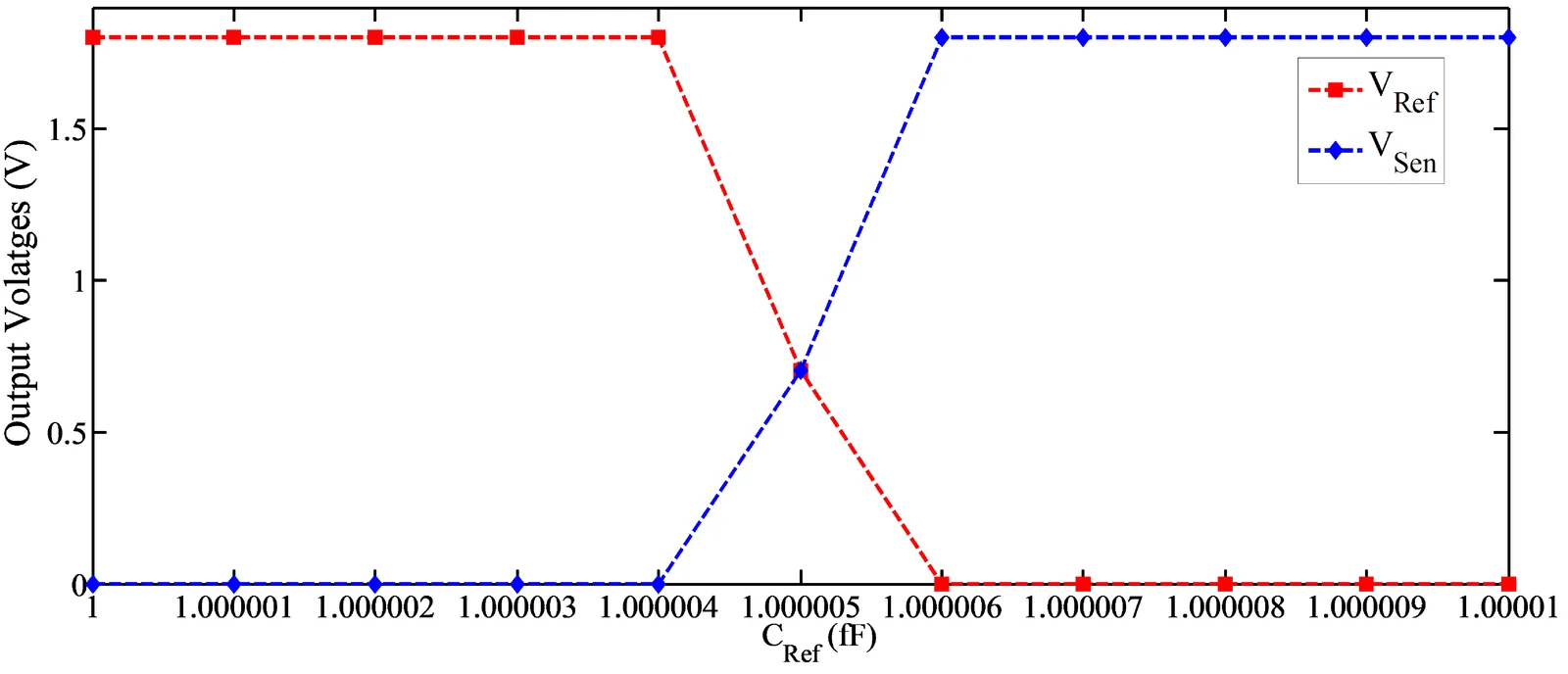
The voltages of $V_{Ref}$ and $V_{Sen}$ versus $C_{Ref}$ at $C_{Sen}$ of 1.000005 fF.

As previously mentioned, the proposed capacitance detection method determines the value of $C_{Sen}$ by adjusting $C_{Ref}$, and monitoring the resulting output voltages. To investigate this, $C_{Sen}$ is set to 1.000005 fF, and $C_{Ref}$ is swept from 1.000001 fF to 1.00001 fF with a step of 1 zF. Then the average values of $V_{Ref}$ and $V_{Sen}$ during the high period of the clock pulse are obtained, as shown in Figure 7. While $C_{Ref}$ is smaller than $C_{Sen}$, $V_{Sen}$ remains at 0 V and $V_{Ref}$ is latched at 1.8 V. When the two capacitances are equal amounts, both $V_{Ref}$ and $V_{Sen}$ are approximately 0.7 V. After $C_{Ref}$ is swept beyond the value of $C_{Sen}$, the voltage $V_{Sen}$ is latched at 1.8 V and $V_{Ref}$ is latched at 0 V. Consequently, this output transition directly represents the capacitance sensing operation.

**Figure 8:**
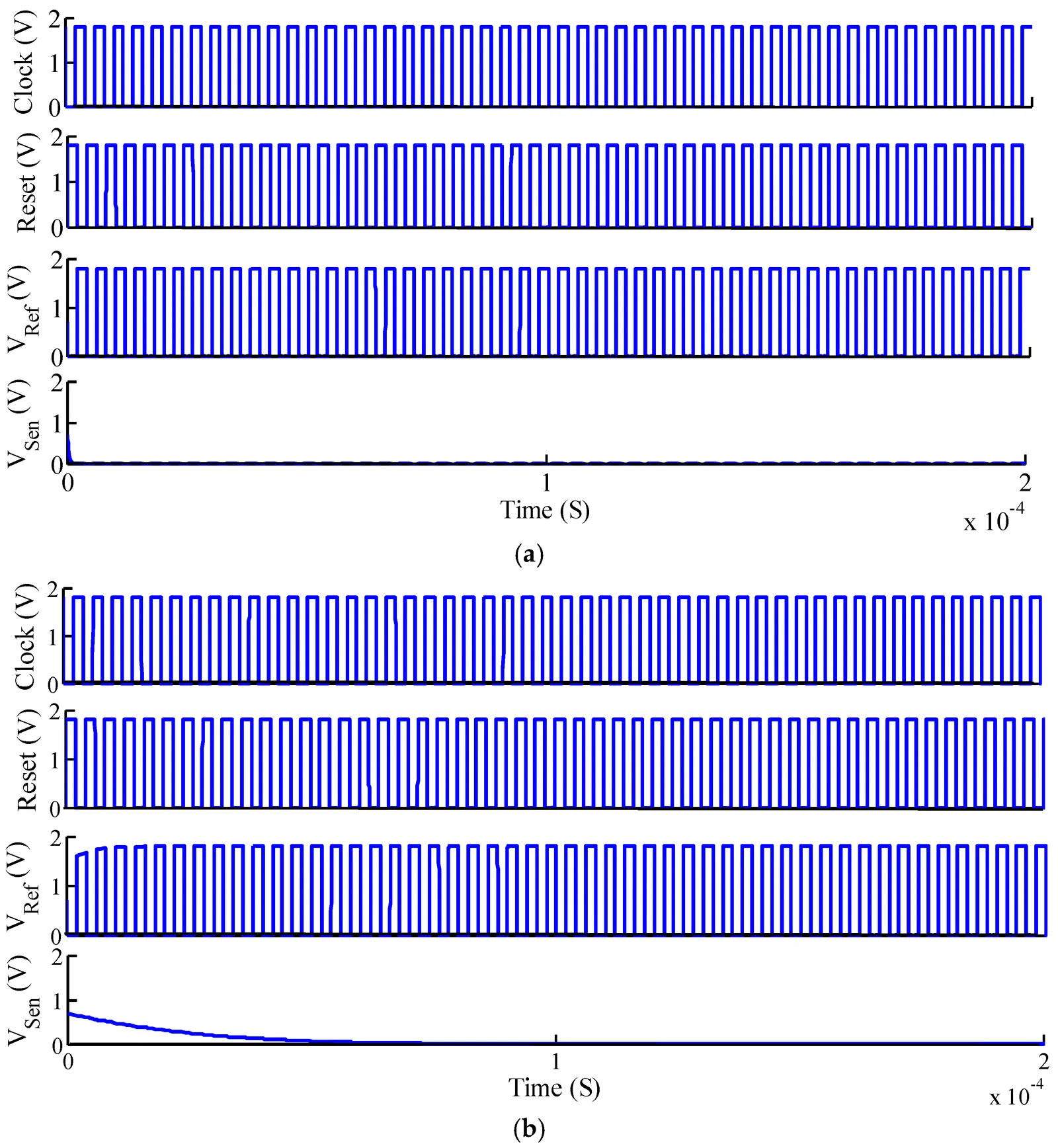
Timing diagram to show capacitance dynamic range (**a**) $C_{Sen}$ = 200 p, (**b**) $C_{Sen}$ = 10 nF.

**Figure 9:**
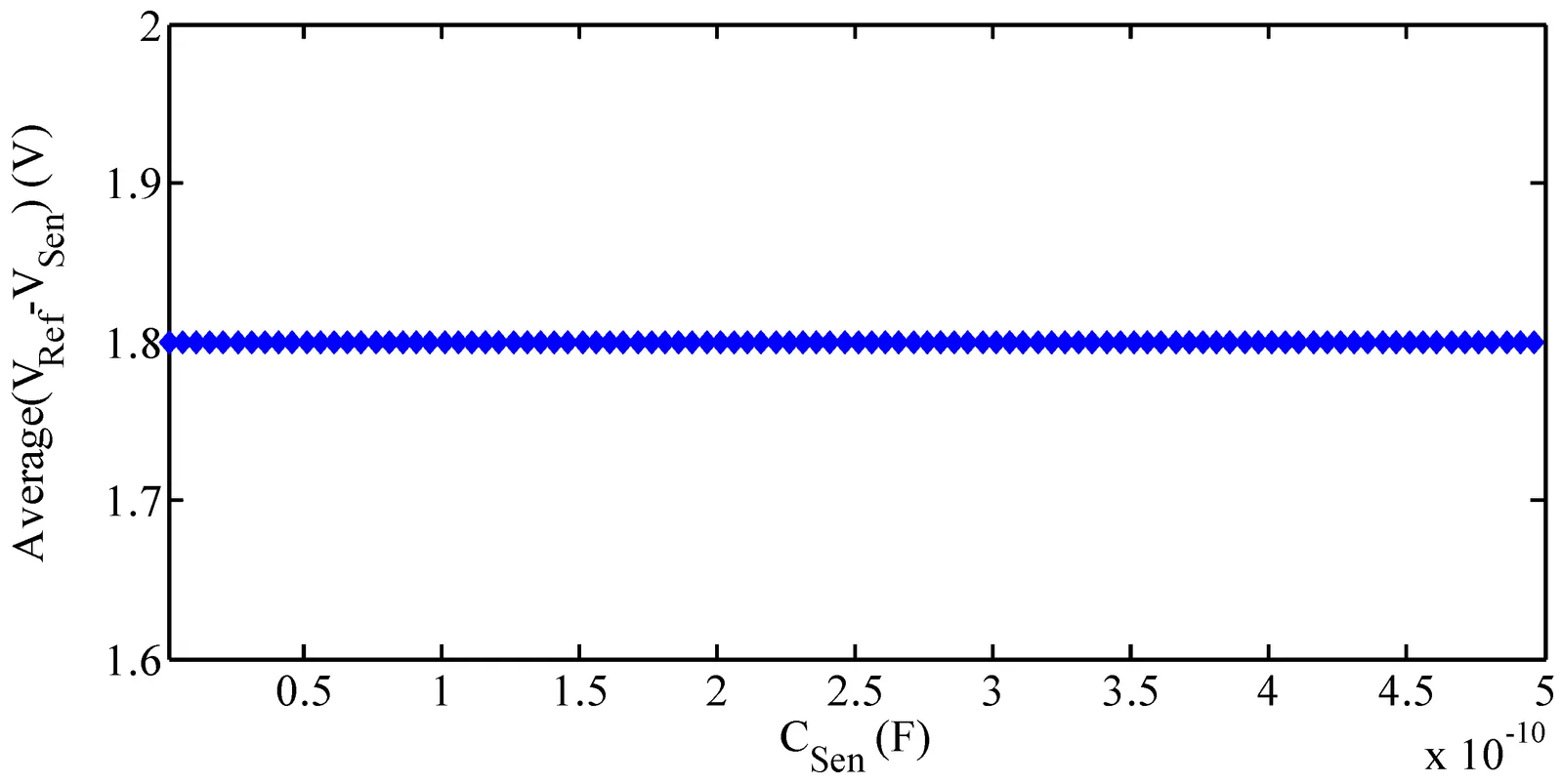
The average difference between $V_{Ref}$ and $V_{Sen}$ versus $C_{Sen}$ swept from 1 pF to 500 pF.

To examine the dynamic range of the introduced CBCD detector, timing diagrams were obtained for $C_{Ref}$ = 1 fF and $C_{Sen}$ values of 200 pF and 10 nF, as shown in Figure 8. Figure 8 shows that larger $C_{Sen}$ values require a longer time for the output node to reach its steady state, while accurate detection is still maintained. Therefore, the introduced CBCD detector can detect capacitance changes from the zF level to values higher than the pF range, resulting in an exceptionally wide dynamic range. Notably, the lower limit is set by parasitic capacitance; below approximately 1 zF, reliable detection is not possible. The upper limit is not constrained by functionality but by the increased decision delay, which scales with capacitance value. The decision delays for ΔC values of 1 zF, 200 pF and 10 nF are 10 ns, 2 µs and 100 µs, respectively. As stated in the manuscript, transient simulations were carried out at a clock frequency of 250 kHz. Further simulations reveal that the proposed CBCD operates correctly up to a maximum clock frequency of 3.5 MHz, above which incorrect detection occurs. The energy per decision is 0.18 µJ. The average value of $V_{Ref}-V_{Sen}$ is obtained when $C_{Sen}$ is swept from 1 pF to 500 pF with a step of 5 pF as depicted in Figure 9.

**Figure 10:**
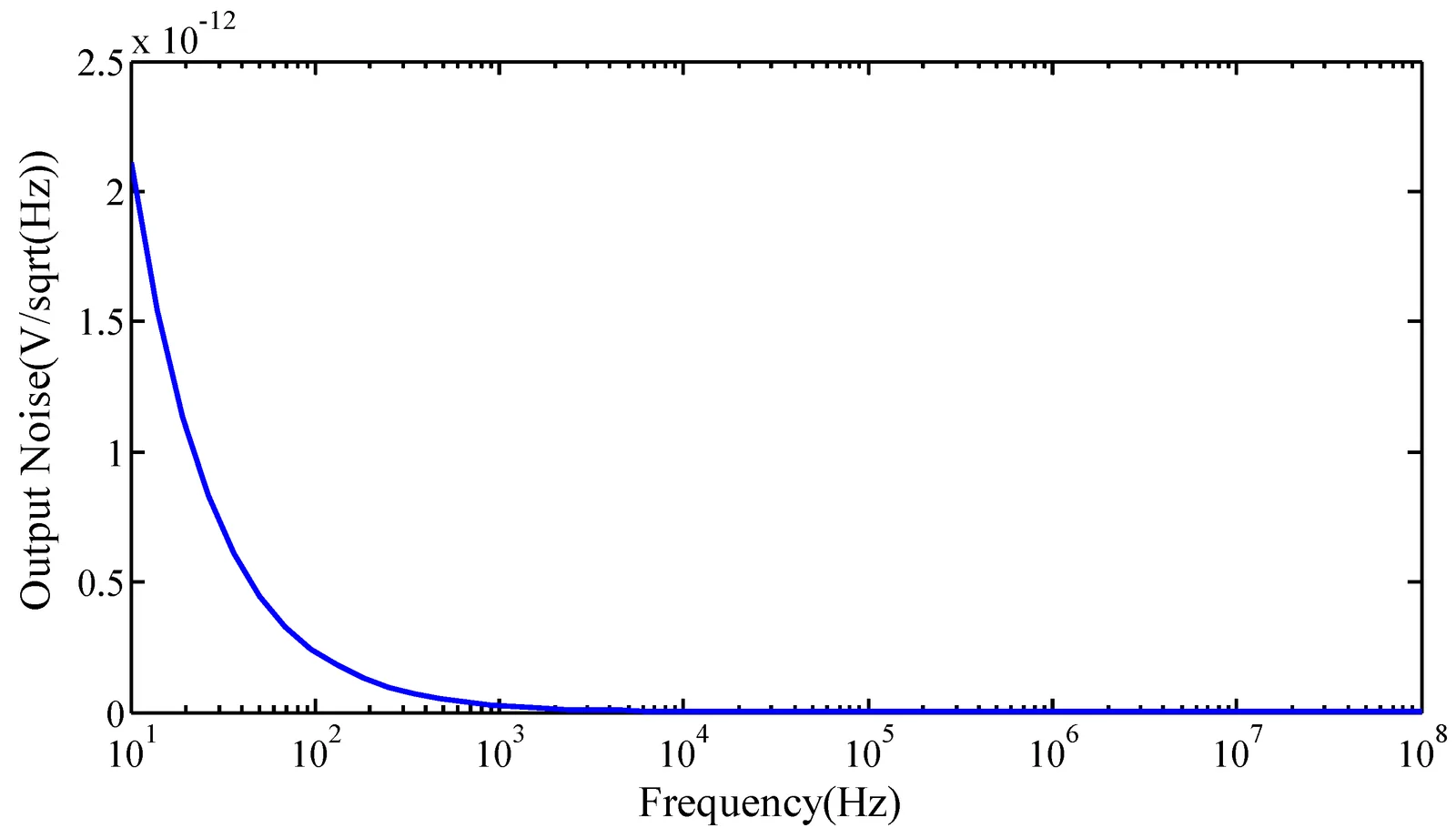
Output noise characteristic of the proposed detector.

To characterize the noise performance of the proposed architecture, frequency-domain noise simulations were performed using Cadence Virtuoso (Spectre RF) with PDK-provided thermal and 1/f noise models. The circuit was simulated at the TT process corner with a nominal temperature of 27 °C. The sensing capacitance difference was set to ΔC = 1 zF. A logarithmic frequency sweep from 10 Hz to 100 MHz was employed, covering the entire bandwidth of interest. The output noise voltage spectral density of the proposed capacitance detector was evaluated as a function of frequency, as shown in Figure 10. The reported noise is approximately 0.24 fV/√Hz at a 1 MHz offset frequency.

**Figure 11:**
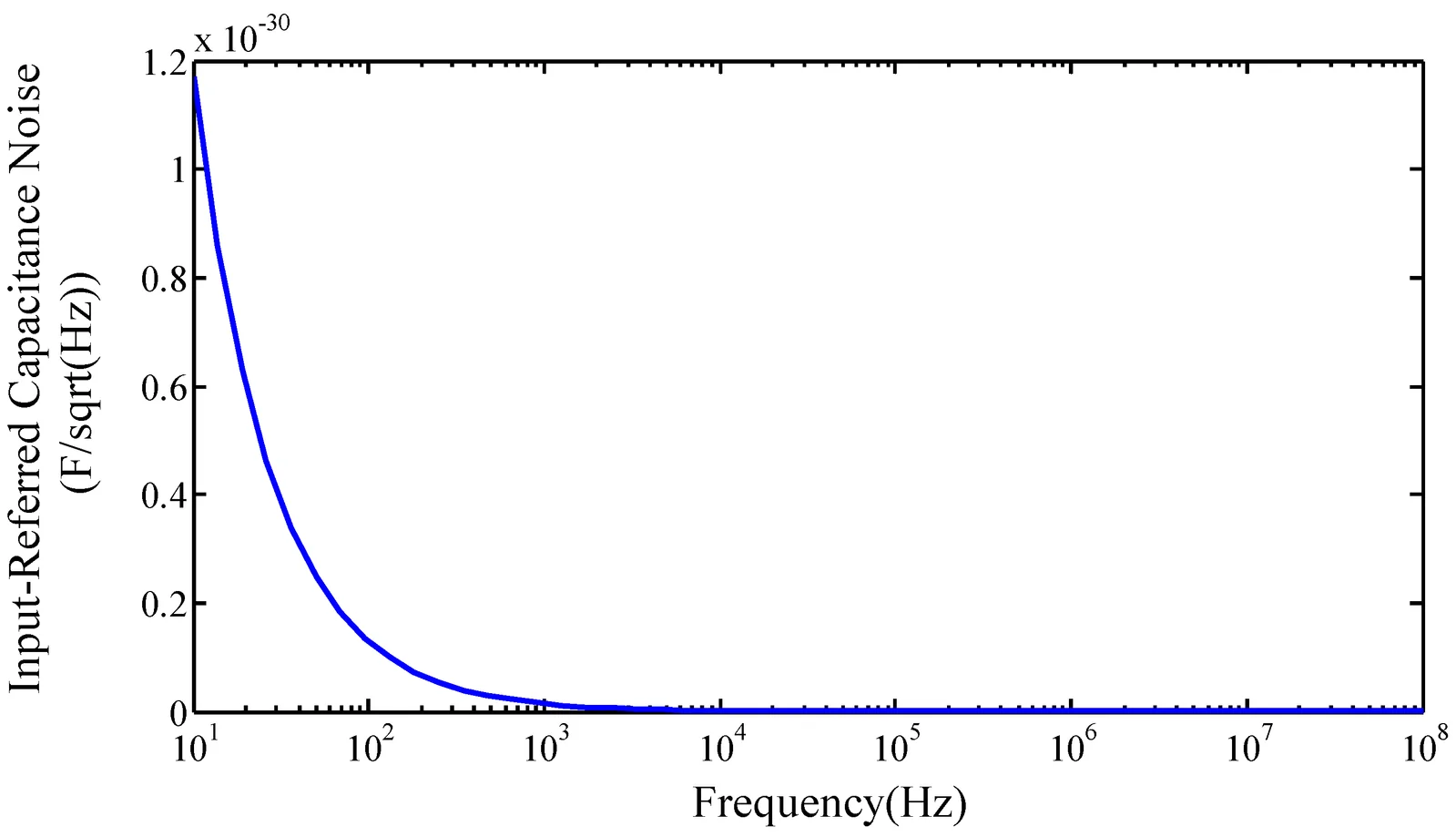
Input-referred capacitance noise characteristic of the proposed detector.

**Figure 12:**
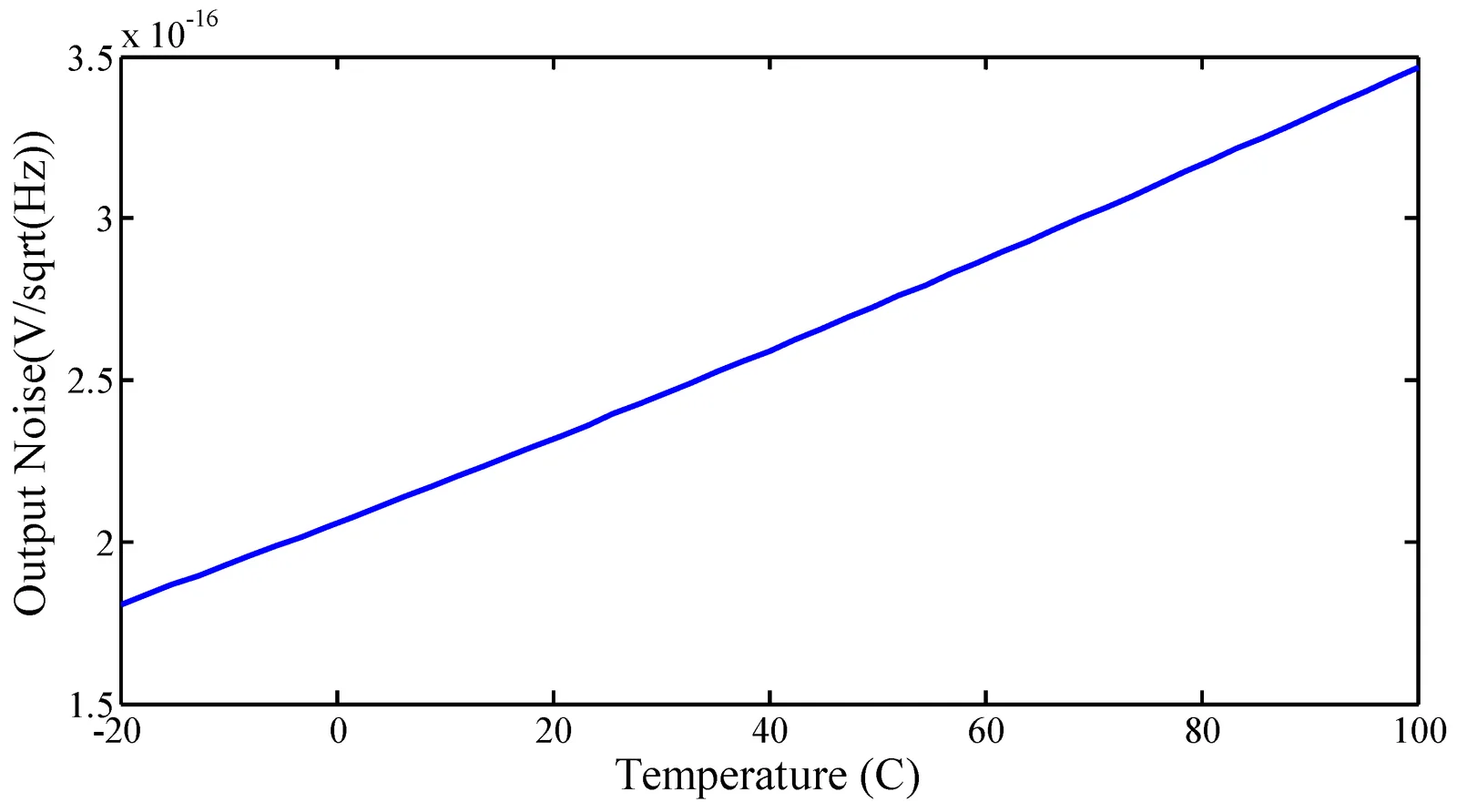
Output noise characteristic at 1 MHz frequency versus temperature of the proposed detector.

Since the core structure is latch-based, the front-end sensing node allows for an equivalent input-referred noise definition. By dividing the output voltage noise spectral density by the transimpedance gain ($∆V_{Ref-Sen}/∆C$) of the front-end stage, an input-referred capacitance noise of 1.33 × 10^−34^ F/√Hz is obtained (see Figure 11). To assess thermal sensitivity, temperature sweeps from −20 °C to 100 °C were performed, as shown in Figure 12. The output noise at 1 MHz varies from 0.18 fV/√Hz at −20 °C to 0.34 fV/√Hz at 100 °C, confirming that the noise level remains around fV/√Hz across the entire operating temperature range.

**Figure 13:**
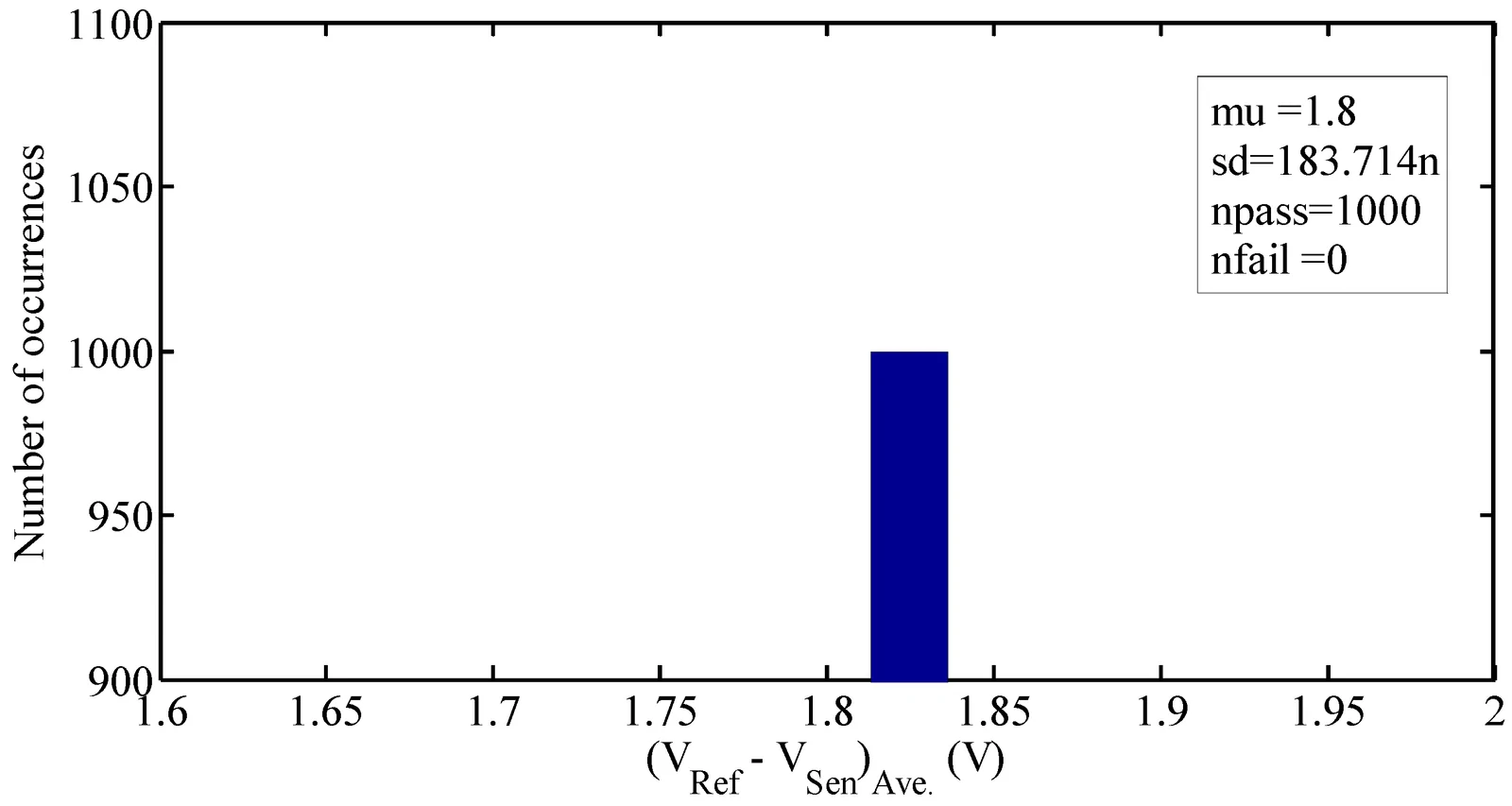
The Monte-Carlo simulation of the average of $V_{Ref}-V_{Sen}$ when $C_{Sen}$ > $C_{Ref}$; (1.000001 fF > 1 fF).

To validate the robustness of the proposed CBCD, its performance was characterized across process and temperature variations. Simulation results, summarized in Table 3, confirm consistent performance across all corners. The output voltage difference remains stable, and the output noise exhibits minimal deviation, demonstrating the circuit’s resilience under these conditions. To further evaluate the robustness of the CBCD against mismatch and process variation, the average value of $V_{Ref}-V_{Sen}$ (when the clock is at a high level) was investigated through Monte Carlo analysis considering process and mismatch statistical variations with 1000 runs, under the condition that $C_{Sen}$ exceeds $C_{Ref}$ by 1 zF. Figure 13 shows that the standard deviation of the average $V_{Ref}-V_{Sen}$ is less than 184 nV. Monte Carlo simulations were performed for multiple ΔC values under temperature variation. The results, summarized in Table 4, confirm that the decision-error probability remains zero for ΔC ≥ 1 zF across all conditions. The proposed CBCD detector maintains reliable operation under process variations and mismatch.

**Figure 14:**
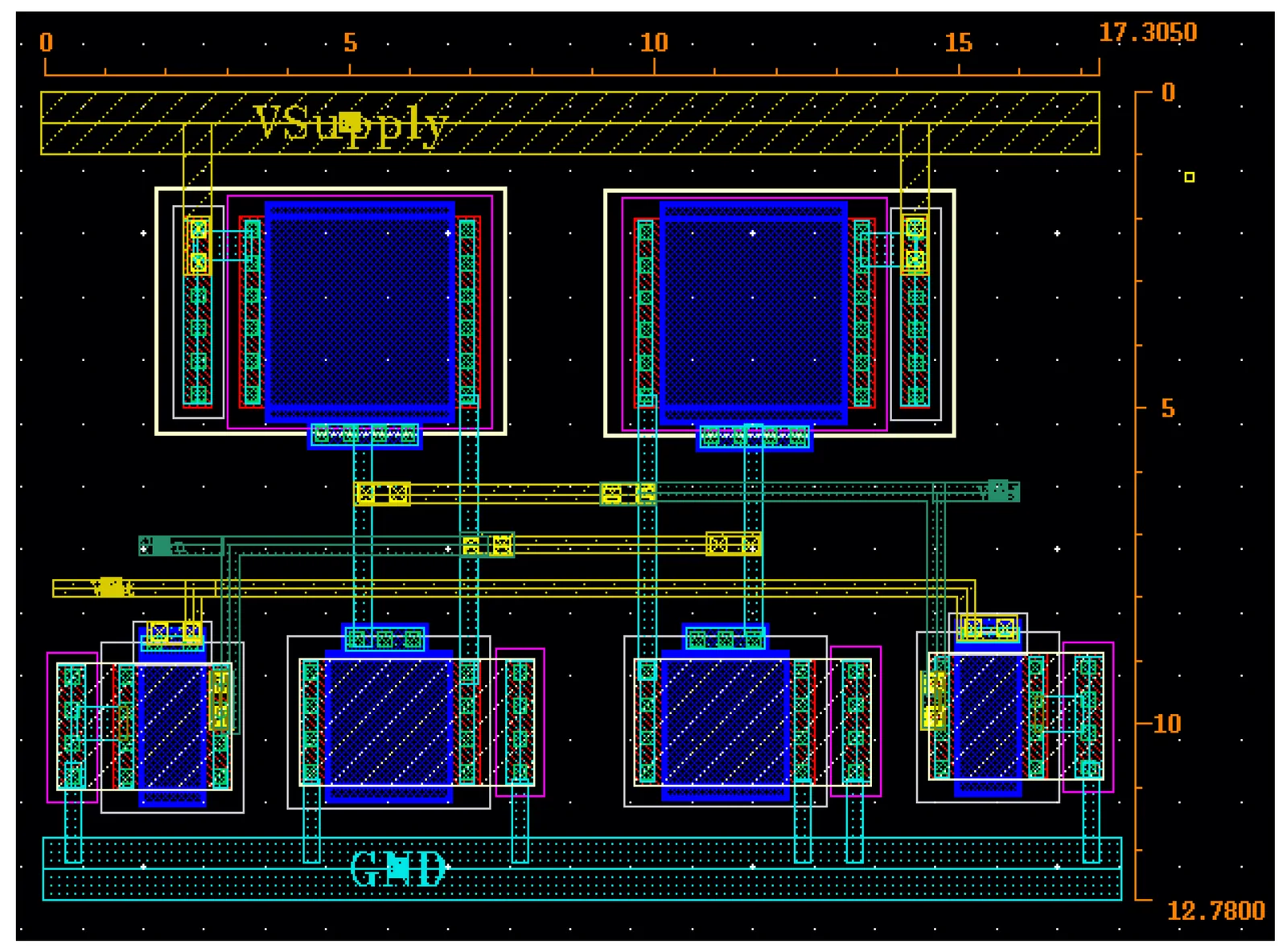
Layout of the proposed CBCD detector.

The layout of the proposed design (without capacitances) is shown in Figure 14, with overall dimensions of 17.31 µm × 12.78 µm. Table 5 presents the design parameters for the proposed CBCD detector.

## 5. Discussion

The results demonstrate that the proposed CBCD enables robust binary capacitance discrimination over an exceptionally wide simulated input range, extending from the zepto-farad regime to the nano-farad regime. Despite producing only a binary output, the detector achieves a simulated decision threshold of 1 zF, demonstrating that high sensitivity can be maintained without requiring quantitative capacitance measurement. By directly converting capacitance imbalance into a digital decision through regenerative positive feedback, the proposed architecture eliminates intermediate readout and conversion stages, thereby reducing circuit complexity and power consumption. These characteristics make the CBCD particularly attractive for applications in which reliable state or event detection is more important than precise capacitance quantification.

Table 6 highlights the architectural principles of integrated CMOS readout interfaces for capacitive detectors. Existing approaches, including oscillator- and CBCM-based circuits, first convert capacitance into a frequency-domain or digital quantity before decision making, thereby separating sensing from the final decision. In contrast, the proposed CBCD uses regenerative positive feedback to compare the sensing and reference capacitances directly within the interface, eliminating intermediate capacitance measurement and combining sensing, comparison, and digitization in a single dynamic stage.

As summarized in Table 6, the RO-based interface in [4] converts capacitance into oscillation frequency, whereas the CBCM-based interfaces in [17,18] first convert capacitance into current before digitization through a ΣΔ ADC. Similarly, the current-mode CBCM reported in [20] translates the capacitance-dependent current into frequency using a current-controlled oscillator followed by frequency counting. Consequently, all previous interfaces require intermediate capacitance conversion and additional signal-processing stages before producing the final output.

The CBCM-based interfaces achieve excellent quantitative sensing performance, including a resolution of 10 aF in [17,18], sensitivities of 250 mV/fF and 350 mV/fF, respectively, and an input dynamic range extending from approximately 2.7 fF in [17] to 10 fF in [18]. The current-mode CBCM in [20] further extends the input dynamic range to 10 fF–1.27 pF with a sensitivity of approximately 400 kHz/fF by introducing a CCO and digital counter. In contrast, the RO-based sensor in [4] provides a 15–21 fF input range with a sensitivity of 46 MHz/fF.

Rather than maximizing quantitative measurement accuracy, the proposed CBCD targets direct binary decision-making. Although it provides only a binary output, it eliminates analog amplification, current integration, analog-to-digital conversion, oscillators, and frequency counters by merging sensing, comparison, and digitization into a single regenerative stage. This architectural simplification enables a simulated decision threshold of 1 zF together with negligible power consumption of 0.45 µW while maintaining robust operation over a simulated input range extending from the zepto-farad regime to the nano-farad regime.

Therefore, compared with the interfaces summarized in Table 6, the primary contribution of the proposed CBCD is not an incremental improvement in quantitative sensing performance, but rather the introduction of a fundamentally different sensing architecture that directly performs capacitance discrimination without intermediate conversion or post-processing.

Although the proposed architecture offers several advantages, some practical considerations should be acknowledged. As with other differential capacitive sensing approaches, the detection threshold is influenced by mismatch between the sensing and reference branches as well as by parasitic capacitances associated with interconnects and sensor structures. While the presented Monte Carlo, temperature, and process-corner simulations show robust operation under expected variations, future experimental investigations will be required to quantify the impact of parasitic effects, environmental noise, and long-term drift in fabricated implementations.

The compact transistor count and fully digital output of the proposed CBCD architecture significantly reduce system complexity. Since each sensing element can directly generate a binary output without requiring local analog signal-conditioning circuitry, the architecture is well suited for scalable, low-power sensing platforms for distributed biochemical monitoring and edge-intelligent sensing applications.

Overall, the proposed CBCD introduces an alternative sensing paradigm that prioritizes state discrimination over quantitative capacitance measurement. For applications in which binary decisions represent the final system objective, the proposed approach offers a compelling combination of high sensitivity, low complexity, negligible power consumption, and straightforward digital interfacing.

## 6. Conclusions

This paper presented a CMOS CBCD for decision-driven capacitive sensing applications. Unlike conventional capacitive interfaces that are designed to quantify capacitance with high accuracy, the proposed architecture directly converts the relative difference between a sensing capacitance and a reference capacitance into a digital output through regenerative positive feedback. By integrating sensing, comparison, and digitization within a single dynamic latch stage, the CBCD eliminates the need for analog amplification, capacitance-to-voltage conversion, analog-to-digital conversion, and frequency-based readout circuits.

Simulation results demonstrate successful capacitance discrimination with a minimum detectable capacitance difference of 1 zF, while maintaining reliable operation over an exceptionally wide simulated input range extending into the nano-farad regime. Noise, process-corner, and Monte Carlo analyses confirmed robust operation under process variations and device mismatch. Furthermore, the dynamic operating principle enables negligible power consumption and supports compact circuit implementation.

The proposed detector is particularly suitable for applications in which the final system objective is a binary decision rather than precise capacitance quantification, including droplet presence detection, bubble monitoring, threshold-based diagnostics, event detection, and ToE measurements. These results suggest that binary capacitance detection represents a promising alternative sensing paradigm for future low-power and highly integrated LoC systems.

Future work will focus on experimental validation of the proposed architecture, characterization in the presence of sensor parasitic effects and environmental noise, and the development of large-scale detector arrays.

## Figures and Tables

**Table 1:** Comparison of capacitive sensors/detectors.

Ref.	Application	Binary Decision Required?	On-Chip Readout Circuit	Direct Binary Decision?	Final Output	Readout Approach
[5]	DNA biosensor	No	No	No	DNA concentration/hybridization level	Capacitance measurement
[6]	Protein biosensor	No	No	No	Protein characterization	HF-EIS
[4]	CMOS cell sensor array	No	Yes	No	Cell characterization and tracking	RO-based capacitance sensing
[7]	COVID-19 immunosensor	Yes	Yes	No	Positive/Negative diagnosis	Threshold-based capacitance measurement
[8]	Infusion bubble monitoring	Yes	No	No	Bubble present/absent	Capacitance measurement
[9]	Fluidic bubble detection	Yes	No	No	Bubble detection event	Differential capacitive sensing + lock-in detection
[10]	Digital microfluidic routing	Yes	No	No	Droplet position/routing decision	Differential capacitance feedback

COVID-19: coronavirus disease 2019; HF-EIS: high-frequency electrical impedance spectroscopy; RO: ring oscillator.

**Table 2:** Comparison of CMOS capacitive sensors’ readout circuits.

Ref.	Method	CMOS Technology	Application	IDR	Sensitivity	Resolution	Array	Direct Binary Decision	Results (E/S)
[1]	Charge sharing	0.25 µm	Single bacterial cell detection	0.45–57 fF	55 mV/fF	450 aF	16 × 16	No	E
[15]	Charge sharing	0.5 µm	Cell proliferation monitoring	NA	NA	5 fF	28	No	E
[16]	Charge-sensitive amplifier	0.35 µm	Localization of bioparticles	0.42 fF	345 mV/fF	21 aF	320 × 320	No	E
[19]	SC + SAR CDC	0.18 µm	Cancer biomarker detection	~16 pF	NA	4.5 fF	3 × 3	No	E
[13]	Relaxation oscillator CFC	0.35 µm	Dopamine detection	12–700 fF	21 kHz/fF	NA	5 × 5	No	E
[11]	Relaxation oscillator CFC	0.5 µm	DNA detection	330 pF–10 nF	23 Hz/pF	NA	8 × 16	No	E
[12]	Ring oscillator CFC	0.35 µm	Cell proliferation monitoring	12 fF	590 kHz/fF	14.4 aF	4 × 4	No	E
[14]	Lock-in detection	0.35 µm	Airborne particle detection	<1 fF	NA	65 zF	32	No	E
[17]	CBCM + ΣΔ ADC	0.18 µm	Bacteria growth monitoring	~2.7 fF	250 mV/fF	10 aF	3	No	E
[18]	Fully Differential CBCM + ΣΔ ADC	0.35 µm	Cell growth monitoring	10 fF	350 mV/fF	10 aF	8 × 8	No	E
[20]	Current-mode CBCM + CCO + Counter	0.35 µm	Liquid sample monitoring/Life-science sensing	10 fF–1.27 pF	≈400 kHz/fF	416 aF	2	No	E

CBCM: charge-based capacitance measurement; SC: switched-capacitor; SAR: successive-approximation register; CDC: capacitance-to-digital converter; CFC: capacitance-to-frequency converter; ΣΔ ADC: sigma–delta analog-to-digital converter; IDR: input dynamic range; NA: not available, E: Experimental results, S: Simulation results.

**Table 3:** Performance evaluation of the proposed design under process and temperature variations.

Corner	Temperature (°C)	ΔV_(REF,SEN) Ave._ (V)	Output Noise (fV/√(Hz))
**tt**	27	1.8	0.24
**ss**	90	1.8	0.36
**ff**	50	1.8	0.25
**sf**	95	1.8	0.34
**fs**	60	1.8	0.28

**Table 4:** Monte Carlo simulation results across different capacitance differences (ΔC).

ΔC (zF)	Temperature	Std. Dev. of ΔV_(REF,SEN) Ave_ (nV).	Decision Error Rate
**1**	27	183.714	0/1000
**10**	60	183.714	0/1000
**100**	5	183.714	0/1000
**1000**	50	183.714	0/1000

**Table 5:** Design parameters for the CBCD.

Parameters	Values
W_MCP1,2_/L_CrsP1,2_	3 µm/3 µm
W_CrsN1,2_/L_CrsN1,2_	3 µm/3 µm
W_Rst1,2_/L_Rst1,2_	2 µm/1 µm

**Table 6:** Comparison of capacitive decision interfaces.

Ref.	Technique	Intermediate Conversion	Decision Mechanism	Technology (Supply Voltage)	Power	IDR	Resolution	Sensitivity	Measurement Time	Readout Complexity
[4]	RO-based CMOS sensor	C → F	Frequency measurement	0.13 µm (1.2 V)	459.3 μW	15 fF–21 fF	-	46 MHz/fF	Long (counter/frequency averaging)	High
[17]	CBCM + ΣΔ ADC	C → I → D	Digital conversion	0.18 µm (1.8 V)	-	~2.7 fF	10 aF	250 mV/fF	Long (ΣΔ conversion)	High
[18]	Fully differential CBCM	C → I → D	Digital conversion	0.35 µm (3.3 V)	-	10 fF	10 aF	350 mV/fF	Long (ΣΔ conversion)	High
[20]	CBCM + CCO + Counter	C → I → F	Frequency counting	0.35 µm (3.3 V)	-	10 fF–1.27 pF	416 aF	≈400 kHz/fF	Long (counter integration)	High
This work	CBCD	None	Regenerative sensing	0.18 µm (1.8 V)	0.45 µW	>1 zF	1 zF	-	Very short (single clock cycle)	Low

C: Capacitance, F: Frequency, I: Current, D: Digital, IDR: Input dynamic range.

## Data Availability

Data are contained within the article. Further inquiries can be directed to the corresponding authors.

## Abbreviations

The following abbreviations are used in this manuscript:

ADC	Analog-to-Digital Converter
CBCD	Cross-Coupled-Based Capacitance Detector
CBCM	Charge-Based Capacitance Measurement
CCO	Current-Controlled Oscillator
CDC	Capacitance-to-Digital Converter
CFC	Capacitance-to-Frequency Converter
CMOS	Complementary Metal-Oxide-Semiconductor
COVID-19	Coronavirus Disease 2019
CSA	Charge-Sensitive Amplifier
CVC	Capacitance-to-Voltage Converter
DAQ	Data Acquisition
DMF	Digital Microfluidics
EIS	Electrical Impedance Spectroscopy
EWOD	Electrowetting-on-Dielectric
HF-EIS	High-Frequency Electrical Impedance Spectroscopy
IDE	Interdigitated Electrode
IDR	Input Dynamic Range
LoC	Lab-on-Chip
MEMS	Microelectromechanical Systems
NMOS	N-Type Metal-Oxide-Semiconductor
PCB	Printed Circuit Board
PID	Proportional-Integral-Derivative
PLL	Phase-Locked Loop
PMOS	P-Type Metal-Oxide-Semiconductor
PVT	Process, Voltage, and Temperature
RO	Ring Oscillator
SAR	Successive Approximation Register
SL	Screen Length
ToE	Time of Evaporation
ΣΔ ADC	Sigma–Delta Analog-to-Digital Converter
zF	Zeptofarad
aF	Attofarad
fF	Femtofarad
